# Fabrication and Study of Micro Monolithic Tungsten Ball Tips for Micro/Nano-CMM Probes †

**DOI:** 10.3390/mi9030133

**Published:** 2018-03-19

**Authors:** Ruijun Li, Chen Chen, Kuangchao Fan, Zhiwei Wang, Fangfang Liu, Qiangxian Huang

**Affiliations:** 1 School of Instrument Science and Opto-Electric Engineering, Hefei University of Technology, Hefei 230009, China; C.Chen@mail.hfut.edu.cn (C.C.); fan@ntu.edu.tw (K.F.); kevin.wang@zoom.us (Z.W.); liuff@hfut.edu.cn (F.L.); huangqx@hfut.edu.cn (Q.H.); 2 School of Mechanical Engineering, Daliang University of Technology, Daliang 116024, China

**Keywords:** monolithic tungsten stylus, microball tip, arc discharge, coordinate measuring machine (CMM) probe

## Abstract

Micro ball tips with high precision, small diameter, and high stiffness stems are required to measure microstructures with high aspect ratio. Existing ball tips cannot meet such demands because of their weak qualities. This study used an arc-discharge melting method to fabricate a micro monolithic tungsten ball tip on a tungsten stylus. The principles of arc discharge and surface tension phenomenon were introduced. The experimental setup was designed and established. Appropriate process parameters, such as impulse voltage, electro discharge time, and discharge gap were determined. Experimental results showed that a ball tip of approximately 60 µm in diameter with less than 0.6 µm roundness error and 0.6 µm center offset could be realized on a 100 µm-diameter tungsten wire. The fabricated micro ball tip was installed on a homemade probe, touched by high-precision gauge blocks in different directions. A repeatability of 41 nm (*K* = 2) was obtained. Several interesting phenomena in the ball-forming process were also discussed. The proposed method could be used to fabricate a monolithic probe ball tip, which is necessary for measuring microstructures.

## 1. Introduction

**Figure 1:**
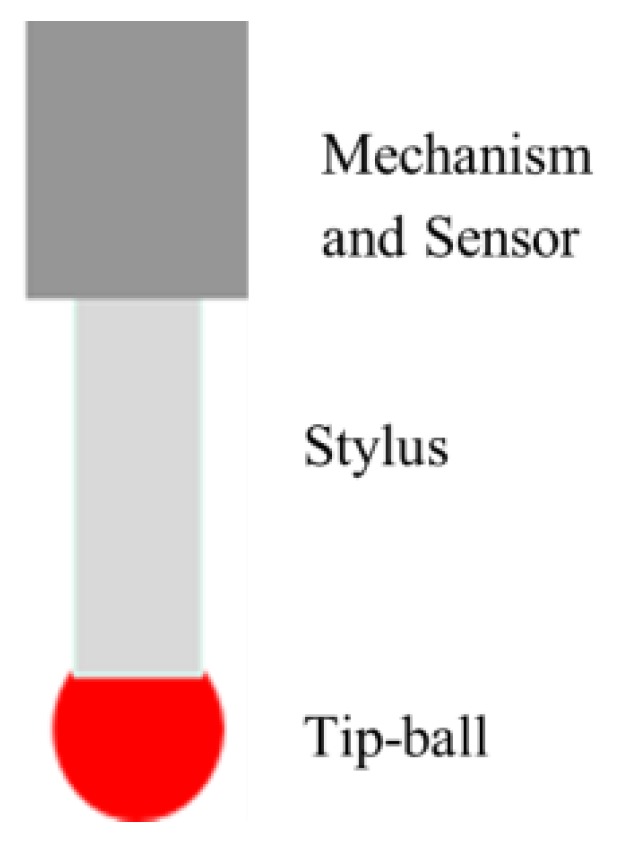
Schematic of a traditional probe.

Various micro/nanostructures or components with geometrical size of nearly on the micro and nanoscales can be produced by micro/nano manufacture technology. Highly accurate micro-3D measurements are required given that the accuracy of miniature products increases. Non-contact optical instruments cannot measure features with high aspect ratio, such as deep holes, lateral walls, and microgrooves [1]. However, the micro/nano-coordinate measuring machines (CMMs) can satisfy these requirements. The contact trigger probe, which is the basic component of micro/nano-CMMs, directly determines the measuring accuracy of the micro/nano-CMMs. The schematic of a traditional probe is shown in Figure 1. To achieve high-precision metrology of micro/nano-CMMs, the probing head requires a highly precise ball-ended stylus tip with accuracy in the submicron order. 

Common probe ball tips are made of ruby, glass, and alloyed steel. The ball tip and probe stylus (or shaft) are fabricated separately. The probe shaft is then inserted into the bore of the ball tip and fixed with adhesive. However, the micro ruby probe ball tip with diameters between 120 and 300 µm is the current state-of-the-art device [2]. The roundness error and center offset of the ball to the stem cannot be proportionally downscaled by the gluing process as the ball becomes even small. The monolithic fiber ball tip is very crisp, soft, and easily damaged. Tungsten exhibits high hardness, abrasion resistance, and toughness [3]. The Moh’s hardness rating of tungsten is 7.5. Therefore, the fabrication of an acceptable monolithic tungsten probe stylus is necessary to avoid the above-mentioned problems.

Fusing the stem end to form a microball tip can minimize the errors introduced by the traditional assembly process. Ji et al. (2005 and 2009) fabricated Bragg fiber tips with a diameter of 200–400 μm by the fiber fusion method [4,5]. Fan et al. (2010) fabricated monolithic fiber tips approximately 300 μm in diameter and with 6 μm roundness error [6]. However, fiber is extremely crisp and easy to be destroyed, and a hard sphere material is necessary given the wear of the microsphere surface due to the contact or scanning force [7]. Sheu (2005 and 2010) developed a wire electro discharge grinding technology to fabricate the tip sphere on a tungsten stem. However, high-precision surface and sphericity of the ball tip are difficult to achieve [8,9]. Therefore, a new arc discharge technique was proposed in a conference to fabricate ball tip on the tungsten wire by the author’s group; as a result, a tungsten ball tip with 1.1 μm roundness and 1.6 μm center offset is fabricated [10]. The fabricating success rate is extremely low, and the ball tip quality is not good enough. The wire is used as one of the electrodes. Using a better electro discharge generator and optimized discharging parameters, the experimental results improve markedly in the current study. A microball tip of approximately 60 µm diameter with a roundness error and center offset of 0.6 µm can be achieved from a 100 µm-diameter tungsten wire by appropriately controlling the process parameters, including the discharge voltage, time, and gap.

## 2. Principles and Setups 

### 2.1. Experimental Principle

#### 2.1.1. Arc Discharge Principle

Electrical breakdown phenomenon will happen when a sufficiently high voltage is applied to the two electrodes. The nonconductive media (such as air) between the electrodes will exhibit a current though, which produces an ongoing plasma discharge. Discharging arc is a charged fluid involved in the fields of heat, electric, magnetic, and fluid. Therefore, the properties of arc can be analyzed by the theory of fluid dynamics.

**Figure 2:**
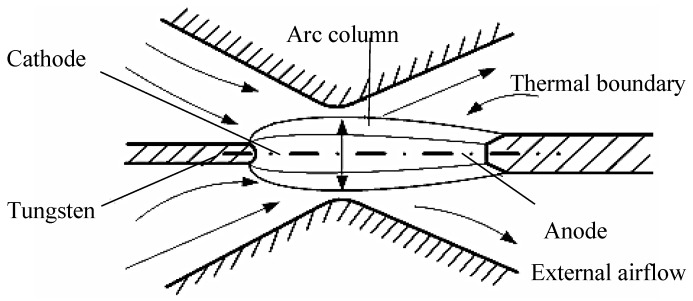
Schematic of arc discharging model.

Figure 2 shows the schematic of an electric arc [10]. According to the physical properties, the electric arc can be divided into three sections: cathode area, anode area, and arc column. The arc closed to the cathode and anode ends are called cathode and anode areas, respectively, whereas the middle section is called the arc column. The border of arc column is called the thermal boundary, and the non-arc column region is called external airflow [11]. The following assumptions are adopted for simplicity: (1) the arc is in the local thermal dynamic equilibrium state; (2) the arc is stable, continuous, and symmetrical and is optically thin and laminar in the state; (3) the heat loss due to viscous effect is negligible. Arc behavior model is controlled by the following equations [12]. Mass continuity equation:

(1) $$\nabla \cdot \vec{\nu }=0$$

Momentum equation:

(2) $$\frac{\partial \vec{\nu }}{\partial t}+\vec{\nu }\cdot \nabla \vec{\nu }=-\frac{1}{\rho }\nabla P+\nu_{e}\nabla^{2}\vec{\nu }-\vec{J}\times \vec{B}+\vec{g}$$

Energy equation:

(3) $$\frac{\partial h}{\partial t}+\vec{\nu }\cdot \nabla h=k\nabla^{2}T+\frac{\vec{J}\cdot \vec{J}}{\sigma }$$

In the equations above, $\vec{\nu }$ is the fluid velocity, *P* is the hydrodynamic pressure, $\vec{J}$ is the current density, $\vec{B}$ is the magnetic field, *T* is the temperature, and *h* is the enthalpy. Material properties include density ρ, effective-viscosity *ν*_e_, kinematic viscosity *ν*, thermal conductivity *k*, and electrical conductivity σ. The permeability of free space $\mu_{0}=4\pi \times 10^{-7}$ Hm^−1^, and the gravitational acceleration *g* is 9.8 m·s^−2^.

On the basis of magnetohydrodynamics theory, the research team of University of Michigan and Southern Methodist University built an arc mathematical model and obtained the temperature distribution model of arc plasma by numerical simulation. The temperature distribution of the arc plasma is intuitively expressed: (1) the heat dissipation in the periphery of the arc is fast. The distribution of radial temperature is the highest on the axis of the arc column, and the temperature decreases gradually along the radial direction [11,12]; (2) the temperature of the two electrodes is higher than that of the arc column. The melting point of tungsten is approximately 3410 °C. The high temperature zone should be used to melt the wire, to save energy.

#### 2.1.2. Surface Tension

**Figure 3:**
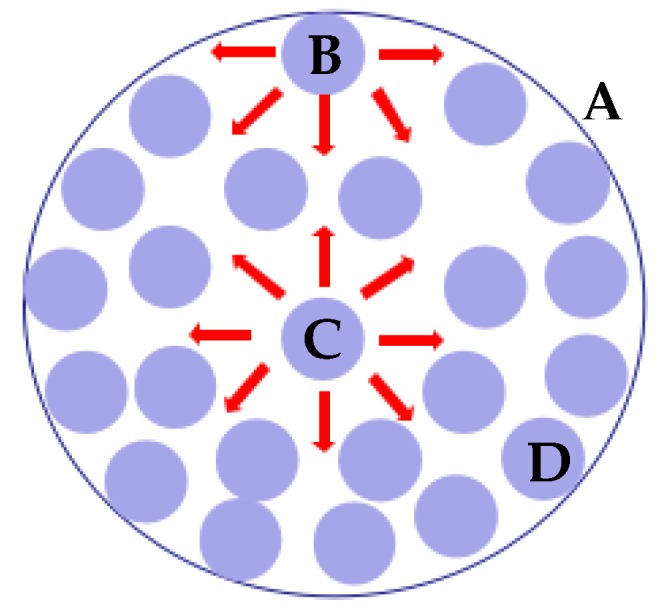
Schematic of surface tension. A: Interface between liquid droplets and air; B: Surface molecule is pulled inward; C: Inner molecule is pulled in all directions; D: Liquid molecules.

The cohesive forces among liquid molecules are responsible for the surface tension [13,14]. In the bulk of the liquid, each molecule is pulled equally in every direction by the neighboring liquid molecules, thereby resulting in a net force of zero. The molecules at the surface are not the same on all sides and are therefore pulled inward. Thus, some internal pressures are created, and liquid surfaces are forced to contract the minimal area. The schematic of surface tension is shown in Figure 3.

### 2.2. Experimental Materials 

Copper, molybdenum, and tungsten are commonly used for electric processing, but the melting and the gasification points of copper are low. Thus, copper is easily gasified when the discharge is carried out. The melting and gasification points of molybdenum are relatively high but are still lower than that of tungsten. Comparisons and analyses for the thermal conductivity and the melting and gasification points of various electrode materials show that tungsten can easily form a ball tip. The current study adopted a fine tungsten wire with a diameter of 100 µm.

### 2.3. Experimental Setup 

**Figure 4:**
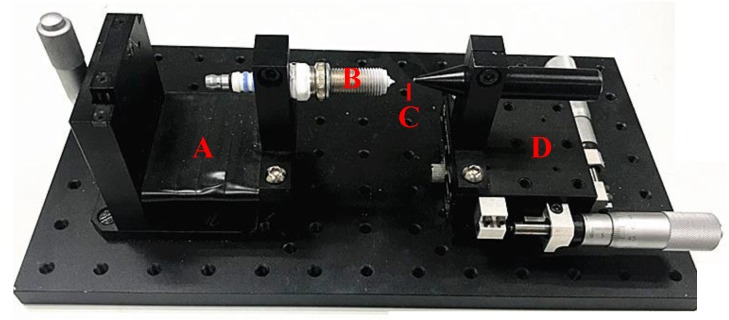
Photo of the discharging device. A: Z-stage; B: Spark plug; C: Tungsten wire; D: Two-dimensional X-Y stage.

The arc discharging device developed is shown in Figure 4. The tungsten wire of 100 μm diameter is normally in a curved shape. In this study, a hollow tube with approximate inner diameter was used to insert the wire in for ensuring the straightness and stiffness of the wire. The front tip of a spark plug was treated as the anode and the tungsten wire as the cathode. The generated heat was sufficiently high to melt the end of the tungsten wire while absorbing the arc discharging power. A three-dimensional (3D) translation stage composed of a two-dimensional (2D) X-Y stage and a vertical Z-stage (all with 20 μm resolution) was used to adjust the alignment and distance between the tungsten tip and the electrode. The tungsten tip and the electrode were fixed on the X-Y stage and Z-stage, respectively. First, the tungsten tip contacted with the electrode tip to keep their alignment by adjusting the two stages. Then, the electrode was kept still, and the electrode tip was treated as a zero point. The distance between the tungsten tip and the electrode could be adjusted and measured by the stage, in line with the electrode’s direction. Oxidation phenomenon occurred when the melted tungsten was exposed in the air, thereby causing heavy smoke. The experimental setup was placed in an acrylic shelter and filled up with inert gas of argon to protect from atmospheric contamination and avoid sparkling smoke. A high-voltage pulse generator (voltage: 0–10 kV, frequency: 5–500 Hz, current: 0–10 mA) was used to control the discharging intensity, and square wave was generated when discharging. A timer was employed to control the fabrication time. The entire setup is economical and flexible to configure.

### 2.4. Profile Measurement

The 2D diameter, roundness error, and center offset were selected as the quality evaluation parameters of the ball tips. The 2D center offset is the distance between the center of the fitting circle and the central line of the tungsten wire. To fit a circle, at least four measuring points of the tungsten ball tip should be selected and analyzed by the least squares circle (LSC) method. In practice of the contact probe, only the lower semi-sphere of the tip-ball will be contacted. The LSC was therefore evaluated from the lower semi-circle data only. Image processing is effective to satisfy the limitation of the resolution given for the small volume of the microball tips. In this study, the ball tip image was offline measured by an optical image system, ADI-ORS100 by Nanjing Optics Robot Technology, which consists of a complementary metal-oxide semiconductor sensor of 1.3 million pixels (1280 × 1024), an optical magnification of ×0.7 to ×4.5, and an image magnification of ×28 to ×180. The smallest field of view was set to be approximately 800 μm × 600 μm, which is sufficiently clear to view the ball tip. A subpixel algorithm was used to detect the image at 0.1 pixel and analyze the geometrical errors [2]. The resolution could reach 1 μm. The light source used was white light. 

## 3. Experiments and Results

**Figure 5:**
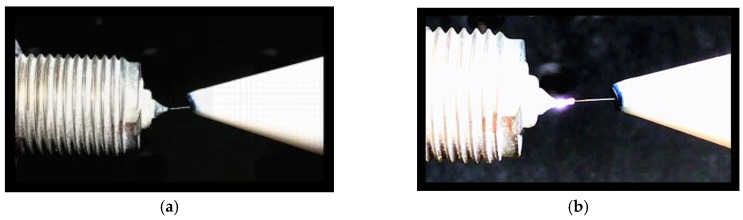
Image of fabricating the tungsten wire ball tip. (**a**) Before discharging; (**b**) During discharging.

The diameter and quality of the tungsten ball tip depend on experimental parameters, and different parameter combinations yield dissimilar fabrication results. The electric arc between the electrodes can provide a high temperature of more than 5000 °C with appropriate parameter combinations. In such a temperature zone, the tip of the tungsten wire is melted, and a microball tip is formed due to the surface tension phenomenon in solidification. A small center offset and a small roundness error indicate satisfactory ball quality. The image of the discharging process, which is shown in Figure 5, was observed in real time.

**Figure 6:**
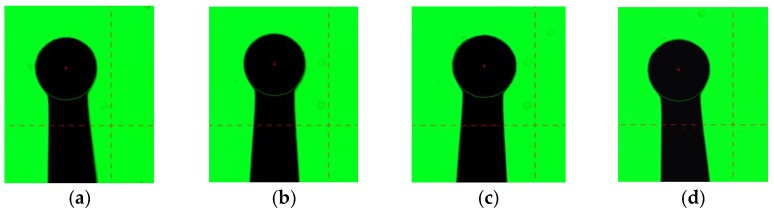
Images of a tungsten ball tip viewed from different aspect angles. (**a**) 0°; (**b**) 90°; (**c**) 180°; (**d**) 270°.

Many discharging parameters influence the fabricated tungsten ball tip. However, the diameter, center offset, and roundness error of the ball tip in this study are mainly determined by impulse voltage, electric discharge duration, and discharge gap according to many experiments. Many different parameter combinations exist to fabricate a ball tip with certain diameter and superior quality. The six parameter combinations used in this study are shown in Table 1. The pulse width is 86.2 ms, which was kept constant. Each parameter combination was used to fabricate the ball tip, which was measured by the optical image system at four angles of view separately (Figure 6). The mean measurement values of four views, which are shown in Table 1, were used to represent the ball tip’s diameter, center offset, and roundness. Therefore, the decimals of 0.25 and 0.75 appear although the resolution of the optical image system is 1 μm. The diameter of the used tungsten wire is 100 μm.

The results in Table 1 reveal that we can fabricate the ball tip with around 60 μm diameter and 1 μm center offset and roundness using the parameter combinations of approximately 10 kV voltage, 1 mm gap, and 6 s time. Actually, a similar ball tip can be fabricated using lower voltage parameter combinations and a 100 μm-diameter tungsten wire. Moreover, better results can be obtained by optimizing the discharging parameters.

A much better ball tip is achieved with discharging parameters of 0.6 kV voltage, 1 mm gap, and 10 s time. This ball tip presents a center offset and roundness of far less than 1 μm. The optical vision-measuring instrument was used in the measurement because of the difficulty in finding an available instrument with a resolution higher than 1 μm. Meanwhile, random error usually exists in measurement. This error could be reduced by repeating the measurement many times and calculating the average value on the basis of error theory. Therefore, to improve the measurement accuracy as much as possible, each geometric size of this tip ball was measured three times in every view. The average values thus obtained were used as the final measurement results (Table 2). Sub micrometers appear when calculating the average value. As shown in Table 2, the center offset and roundness of this ball tip are less than 0.6 μm.

The current experimental results prove the feasibility of fabricating monolithic tungsten ball tip by changing the three parameters. Other fabrication parameters, such as pulse width and raw material, are not yet studied. Thus, further studies are required to examine their influences.

## 4. Application

**Figure 7:**
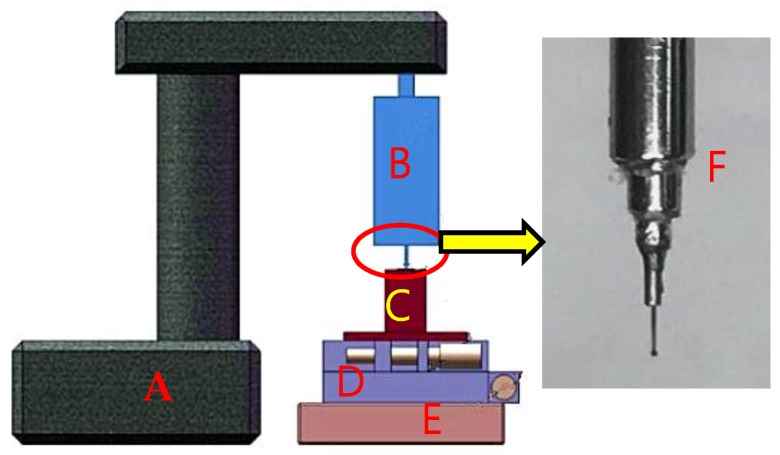
Sketch of the experimental setup. A: Stable stand frame, B: Probe, C: Fixture of the four gauge blocks, D: Two-dimensional high-precision stage, E: Physik Instrumente (PI) stage, F: Micro monolithic tungsten tip.

An experimental setup shown in Figure 7 was constructed to verify the practicability of the fabricated micro ball tips. A micro-monolithic tungsten tip glued to a needle tube was installed on a homemade contact probe [15]. The probe was installed on a discarded angle instrument’s stand frame, which is made up of cast iron and exhibits a weight of more than 20 kg, to improve stability. A high-precision 3D nano-positioning stage (Physik Instrumente (PI), model P-561.3CD with 2 nm repeatability and 100 µm travels) was used as a displacement reference, which was produced by Physical Instrument, Karlsruhe, Germany. A 2 mm × 2 mm square hole established by four 0 grade gauge blocks were used to contact the ball tip from different horizontal directions. A 3D manual adjusted stage was used to change the initial position of the square hole. The probe’s repeatability along the X+, X−, Y+, Y−, and Z directions was tested separately using this experimental setup. The measurement procedure in each direction is similar. The first step is to let the surface of one of a gauge block close to the probe ball tip as much as possible by adjusting the 3D manual stage. The second step is to move the PI stage to find the trigger point by the “double trigger method” [16]. The third step is to push the probe ball tip to 1 μm displacement and record the probe’s output. The last step is to move the block module back to 1 μm displacement. The procedure was repeated ten times. A total of 10 probe values are obtained in each direction, and 10 residual errors are obtained by subtracting their mean value using the 10 probe values in each direction. Table 3 shows the residual errors of the test results in each direction.

## 5. Discussion

**Figure 8:**
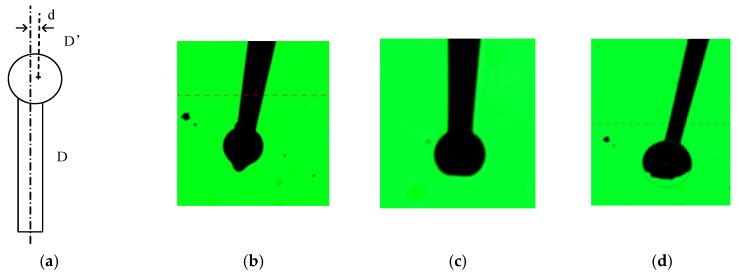
(**a**) Schematic of the tip ball center offset; (**b**–**d**) Images of typical poor tungsten tips.

During solidification, the drift of the ball due to gravity effects causes the offset d of the ball from central line DD’ of the tungsten stem (Figure 8a). In the previous research of ball tip fabrication on optical fiber, the fiber was placed at the middle position of two electrodes in horizontal. The fiber could be rotated during the process to compensate for the gravity [14]. In the present study, the tungsten wire was treated as the cathode, which could not be easily rotated by an appropriate mechanism that must isolate high voltage pulse generator. Therefore, we preferred to deal with the problem by trying to fit the center line of the electrodes and adjusting the discharge gap and other parameters.

If the fabrication process is not effectively controlled, then the distorted shape of the micro ball tip will be formed because of the vaporization or the lack of surface tension. Figure 8b–d show three typical shapes of the micro ball tips. Figure 8b shows that, when the instantaneous energy supply caused by electric arc is slightly large, the front tip of the formed sphere will melt again. Then, the melt tungsten will expand longitudinally and form a prominent raised section. Figure 8c shows that, when the instantaneous energy supply caused by electric arc is large, a large portion of the front tip of the formed sphere will melt and evaporate. Then, the flat head appears at the front tip of the sphere. Figure 8d shows that, when the instantaneous energy supply caused by electric arc is extremely large, melt vaporization will occur. Meanwhile, the probe ball is distinctly mutilated. In general, the high temperature and instantaneous energy caused by electric arc will result in the melting at the front tip of the tungsten electrode. This condition can eventually result in vaporizing if ineffectively controlled. Unbalanced thermal conductivity and solidification force will lead to a high variation in the sphericity and roundness of the ball tip. Fortunately, the experimental results shown in Table 1 reveal that the microball tip with improved roundness can be fabricated by optimizing the processing parameters. 

**Figure 9:**
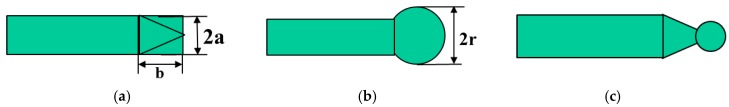
Schematic of the different micro-ball tips. (**a**) Before fabricating; (**b**) Fabricating with sufficient energy; (**c**) Fabricating with insufficient energy.

Interestingly, the tungsten wire tip becomes tapered in shape during the growth of the ball. The tungsten wire and spark plug are coaxially placed. Thus, when the power is switched on, the arc discharge is formed between the spark plug and the tungsten wire. Along the arc cross section, the temperature gradually decreases from the inside to the outside. When the instantaneous energy supply is sufficient, the cylindrical tungsten with a diameter of 2a and a length of b can be melted to form a sphere with a diameter of 2r, as shown in Figure 9a,b. When the instantaneous energy supply is insufficient to melt the cylindrical tungsten with a diameter of 2a and a length of b, the melting process will change and be divided in two steps. Considering the center temperature of the arc is higher than the around temperature, the central part of the tungsten wire melts first other than that of the surrounding part [12]. The molten liquid in the central part expands longitudinally and forms a finer cone than the original tungsten wire. Then, the instantaneous energy supply becomes sufficient for the end of the cone, and a microball with a diameter smaller than the tungsten wire can be formed, as shown in Figure 9c. Such a tapered geometry is beneficial for this experiment to obtain small ball tips. The length and the straightness were measured by the optical measuring instrument, with typical values of 0.73 mm and 1 µm, respectively. If sufficient instantaneous energy is supplied, then large diameter ball tips can be achieved.

## 6. Conclusions

This study used an economical and flexible method based on arc discharge to fabricate a monolithic micro-spherical tip using tungsten wire. The tungsten tip was treated as an electrode to absorb sufficient heat. The high temperature and instantaneous energy caused by the electric arc would melt the front tip of the tungsten wire. The unbalanced thermal conductivity and solidification force would lead to a significant variation in the sphericity and roundness of the ball-ended tip. The experimental results reveal that a spherical tip with approximately 60 µm diameter, less than 0.6 µm roundness error, and 0.6 µm center offset can be achieved by a 100 µm-diameter tungsten wire with appropriate process parameters. Further studies will focus on the improvement of the ball tip quality by investigating additional parameters. The experimental results also verify that the fabricated ball tips are suitable for the application on contact probe of micro/nano-CMMs.

## Figures and Tables

**Table 1:** Experimental parameters and results.

No.	Voltage (kV)	Gap (mm)	Time (s)	Diameter (µm)	Center Offset (µm)	Roundness Error (µm)
1	9	1	6	64.0	1.25	1.0
2	9	1	6	65.5	2.0	1.25
3	10	1	5	71.75	0.25	1.25
4	10	1	6	71.0	1.0	0.25
5	10	2	5	62.0	1.0	0.75
6	10	2	6	58.75	0.75	1.0

**Table 2:** Measurement results of the tungsten ball tip at four view angles.

Angle of View/Degrees	0°	90°	180°	270°
Diameter (µm)	69.6	69.8	69.3	68.8
Roundness (µm)	0.1	0.3	0.6	0.1
Center Offset (µm)	0.6	0.5	0.2	0.2

**Table 3:** Results of the trigger test.

Item	Residual Errors (nm)				
	X+	X−	Y+	Y−	Z
1st	−14.8	30.2	12.3	2.1	−6.2
2nd	−8.1	−7.3	40.9	−5.6	−50.4
3rd	−19.1	20.0	0.8	−10.8	4.2
4th	23.0	−8.8	9.0	13.9	−3.6
5th	−2.6	3.0	−18.7	5.9	24.9
6th	9.8	−3.3	−0.9	−5.9	−1.0
7th	18.6	−11.3	−17.2	−12.1	14.5
8th	−15.4	−33.2	2.7	0.5	−3.6
9th	6.0	12.9	−1.3	−8.2	−6.2
10th	2.6	−2.3	−27.6	20.3	27.5
Standard deviation	13.8	16.9	18.2	10.2	20.6
